# Lymph-node-first Kawasaki disease obscured by deep neck infection in an infant

**DOI:** 10.1016/j.idcr.2026.e02636

**Published:** 2026-06-15

**Authors:** Mano Harshadan, Bharathi Elangovan, Jayavardhana Arumugam, Minoti Subhash Kale, Navin Palaniappan Nachiappan

**Affiliations:** Department of Pediatrics, PSG Institute of Medical Sciences and Research, Coimbatore (Affiliated to the Tamil Nadu Dr. M.G.R Medical University), Off Avinashi Road, Peelamedu, Coimbatore, Tamil Nadu 641004, India

**Keywords:** Deep neck infection, Kawasaki disease, Lymph-node-first Kawasaki disease, Coronary aneurysm

## Abstract

Fever, suppurative lymphadenitis, and suppurative parapharyngeal and mediastinal abscesses are typically indicative of deep neck infections. However, in rare instances, such deep neck infections may obscure underlying autoimmune conditions. This can lead to delayed or missed diagnosis with serious consequences. This case report describes the diagnostic uncertainty in a child presenting with suppurative deep neck and mediastinal abscesses concurrently with lymph-node-first Kawasaki disease features. Early clinical suspicion based on partial antibiotic response, timely detection of bilateral coronary artery dilatation and aneurysm, and prompt dual management with abscess drainage and intravenous immunoglobulin led to complete recovery of the child. This case highlights the importance of maintaining a high index of suspicion for Kawasaki disease in children with persistent lymphadenopathy and fever spikes despite intravenous antibiotics, while also recognizing the potential for true deep neck infection.

## Introduction

Kawasaki disease is an acute, febrile, multisystem disorder emerging as the leading cause of acquired heart disease in children under five years with an incidence of 18–25 per 100,000 in the United States. In Northeast Asian nations such as China, Taiwan, South Korea, and Japan, the incidence is 10–30 times higher [1]. It predominantly affects males (male-to-female ratio ∼1.5:1) and has a reported recurrence rate of about 3% with a case fatality rate below 0.1% [2]. The disease presents clinically with high-grade fever lasting at least 5 days, polymorphous rash, non-suppurative cervical lymphadenopathy, conjunctival congestion and mucocutaneous changes [3]. Although typical cases are well recognized, atypical presentations may delay or mislead diagnosis. Lymph-node-first Kawasaki disease is one such uncommonly encountered atypical manifestation, where cervical lymphadenopathy is the first physical presentation. Such patients tend to be older, with an increased risk of cardiovascular complications and a greater likelihood of requiring additional intravenous immunoglobulin (IVIg) treatment [4]. Administering IVIg within the first 10 days of fever significantly reduces the risk of developing coronary artery aneurysm [5]. This case report describes a unique presentation of suppurative deep neck and mediastinal abscesses with underlying lymph-node-first Kawasaki disease, highlighting a diagnostic uncertainty and emphasizing the need for early recognition and timely treatment which led to complete recovery.

## Case presentation

A 10-month-old boy was referred with a history of high-grade fever for 6 days and swelling on the right side of the neck for 4 days. Prior to admission, the child received ceftriaxone and azithromycin along with the antiviral oseltamivir for 5 days at another center (Fig. A). On examination, the child was irritable, febrile (38.33°C), and tachypneic with pallor and periorbital puffiness. He measured 70 cm in length and weighed 8 kg. Respiratory rate was 58 breaths per minute, capillary refill time less than three seconds, and oxygen saturation (SpO₂) 98% on supplemental oxygen. Local examination showed a diffuse, right-sided, 5 × 5 cm neck swelling that was warm, tender and firm, while systemic examination was normal. Laboratory tests showed microcytic hypochromic anemia with neutrophilic leukocytosis, hypoalbuminemia, elevated erythrocyte sedimentation rate (ESR) and positive C-reactive protein (CRP). Other biochemical parameters were normal (Table 1). Serological testing for alternative infectious etiologies, including dengue (NS1, IgM, IgG by CLIA) and scrub typhus (IgM by ELISA), was negative. Ultrasonography (USG) of the neck revealed right-sided cervical lymphadenopathy with multiple conglomerate lymph nodes at II, III and IV cervical stations. Necrosis and abscess formation were noted on a few nodes of the right cervical station. Intravenous vancomycin was added to ceftriaxone for broader gram-positive and gram-negative bacteria coverage. However, only a partial response was observed despite administration of ceftriaxone for 7 days and vancomycin for 48 h. Considering the child’s age, prolonged fever (>5 days), cervical lymphadenopathy, elevated CRP, anemia, leukocytosis, and partial antibiotic response, an alternate presumptive diagnosis of lymph-node-first presentation of Kawasaki disease was considered. However, no polymorphous rash, palmar erythema, conjunctival congestion, or strawberry tongue was noted. Echocardiography revealed a medium aneurysm of the Right Coronary Artery (RCA), dilatation of the Left Main Coronary Artery (LMCA) and a normal Left Anterior Descending Artery (LAD) (Z scores: RCA - 7, LMCA - 2.49, LAD - 0.87). Based on seven days of unexplained fever, CRP ≥ 3 mg/dL, ESR ≥ 40 mm/h, anemia for age, WBC ≥ 15,000/mm³ , albumin ≤ 3 g/dL, and an RCA Z-score ≥ 2.5, the American Heart Association (AHA) 2024 criteria for suspected incomplete Kawasaki disease were fulfilled [1].

Subsequently, treatment with high-dose IVIg (2 g/kg single infusion over 10 h) and aspirin (100 mg/kg/day) was initiated. A deep-seated infection was still under consideration, so ceftriaxone and vancomycin were continued. Computed tomography (CT) scan of the neck and thorax revealed a right parapharyngeal abscess collection measuring 2.4 × 1.2 × 8.0 cm (Transverse x Anteroposterior x Craniocaudal) with a volume of ∼ 12 cc extending into the right carotid space. The suppurative abscess extended from the angle of the mandible to the superior and middle mediastinum, up to the cavoaortic junction or carina (Fig. B). Under ultrasonographic and C-arm guidance, 50 mL of pus was drained from the abscess, which on culture grew Methicillin-resistant Staphylococcus aureus (MRSA) sensitive to vancomycin and clindamycin. However, the final blood culture was sterile. Ceftriaxone was thereby stopped, and vancomycin was continued. During the treatment course, platelet count showed serial elevation, while CRP and ESR declined. The child became afebrile by day 4 of hospitalization. Vancomycin was replaced by linezolid oral suspension from day 10, given its reliable oral bioavailability and tissue penetration, as well as concerns regarding inducible clindamycin resistance. Follow-up echocardiography before discharge showed improvement in coronary artery dilatation and aneurysm. Discharge occurred on day 13 with a prescription for linezolid oral suspension for 4 days and aspirin therapy. Aspirin was later discontinued 6 weeks after discharge. At further review visits, echocardiography demonstrated resolution of coronary artery dilatation and aneurysm, while blood counts showed continued improvement.

## Discussion

The child presented with fever, suppurative lymphadenitis, and suppurative parapharyngeal and mediastinal abscesses, which are typically seen in deep neck infections [6]. However, the incomplete and transient response to antibiotics, along with specific concerning features, raised suspicion for an alternative or concurrent inflammatory process. Incomplete Kawasaki disease was considered in view of compatible laboratory and echocardiographic findings as per the American Heart Association 2024 guidelines, in addition to unexplained fever for 7 days [1]. The presence of microcytic hypochromic anemia in this child may reflect underlying iron deficiency, which could confound its interpretation as a supportive criterion for Kawasaki disease. As Kawasaki disease remains a clinical diagnosis, the possibility that delayed or incomplete source control contributed to the partial antibiotic response cannot be excluded. In addition, pharmacokinetic and pharmacodynamic limitations of intravenous vancomycin, particularly in achieving optimal therapeutic levels in young children, may have also played a role. While the findings fulfilled criteria for incomplete Kawasaki disease, a purely infectious etiology with systemic inflammatory response cannot be definitively excluded, particularly given the severity of MRSA infection and timing of source control.

At this stage, the clinical picture reflected overlapping features of both deep neck infection and incomplete Kawasaki disease, making diagnostic differentiation challenging. Management was consequently directed at both possibilities, with same-day abscess drainage for the deep neck MRSA infection alongside initiation of IVIg for suspected Kawasaki disease. While this dual approach was clinically necessary to mitigate risk, it complicates the retrospective determination of which intervention was primarily responsible for the rapid defervescence. Additionally, clinical improvement following IVIg should be interpreted with caution, as anti-inflammatory effects may also transiently improve infection-associated inflammation.

This case highlights the diagnostic uncertainty when a deep neck infection coexists with features suggestive of Kawasaki disease. The challenge was compounded by the presence of lymphadenopathy with abscess and necrosis formation, which is atypical for Kawasaki disease [3]. This also contrasts with the non-suppurative lymphadenopathy described in lymph-node-first Kawasaki disease by Kanegaye et al. [6]. Additionally, unlike previous reports where typical Kawasaki disease symptoms, such as rashes, developed during hospitalization, this case remained atypical throughout, showing no classical features even in later stages. Kim et al., Huo et al., and Chen et al. [4], [7], [8] reported progressive development of classical features that aided diagnosis in patients with lymph-node-first or atypical Kawasaki disease associated with suppurative lymphadenitis and abscess formation.

Furthermore, coronary artery dilatation and aneurysms have been reported, albeit rarely, in other infectious and inflammatory conditions, including *Mycoplasma pneumoniae*, Epstein-Barr virus infections, and multisystem inflammatory syndrome in children (MIS-C). However, such findings are not well described in localized suppurative infections such as deep neck or mediastinal abscesses, where vascular complications, when present, more commonly involve adjacent vessels such as the carotid artery [9]. In such scenarios, Kawasaki disease is frequently prioritized as a key differential diagnosis and systematically evaluated before considering alternative etiologies [10], [11]. Given the fulfillment of AHA criteria and clinical context, further extensive testing for alternative etiologies was not pursued. In this context, the presence of coronary artery involvement in our patient increases the likelihood of Kawasaki disease as a unifying diagnosis, although it does not entirely exclude the possibility of infection-associated inflammatory vascular changes.

Although a single case, it underscores the importance of maintaining a high index of suspicion for Kawasaki disease in children with persistent lymphadenopathy and fever spikes despite intravenous antibiotics, while also recognizing the potential for true deep neck infection. Echocardiography should be performed early to detect coronary complications in such equivocal presentations and prevent poor outcomes.

## Privacy statement

Privacy rights of the patient have been observed by removing any personal identifying information in the manuscript and figure image.

## CRediT authorship contribution statement

**Mano Harshadan:** Writing – review & editing, Writing – original draft, Conceptualization. **Bharathi Elangovan:** Writing – review & editing, Writing – original draft, Supervision, Project administration, Conceptualization. **Jayavardhana Arumugam:** Writing – review & editing, Writing – original draft, Supervision, Project administration, Conceptualization. **Minoti Subhash Kale:** Writing – review & editing, Writing – original draft, Supervision, Project administration, Conceptualization. **Navin Palaniappan Nachiappan:** Writing – review & editing, Writing – original draft, Conceptualization.

## Informed consent

Written informed consent was obtained from the patient for publication of this case report and accompanying images. A copy of the written consent is available for review by the Editor-in-Chief of this journal on request.

## Institutional Human Ethics Committee approval statement

Approved by PSG Institute of Medical Sciences & Research Institutional Human Ethics Committee (Recognized by SIDCER, WHO) vide reference no. PSG/IHEC/2025/Appr/Exp/036 on 08/02/2025.

## Declaration of Generative AI and AI-assisted technologies in the writing process

During the preparation of this work the author(s) used ChatGPT in order to improve readability and language of the manuscript. After using this tool/service, the author(s) reviewed and edited the content as needed and take(s) full responsibility for the content of the published article.

## Funding

This research did not receive any specific grant from funding agencies in the public, commercial, or not for-profit sectors.

## Declaration of Competing Interest

The authors declare that they have no known competing financial interests or personal relationships that could have appeared to influence the work reported in this paper.

## Figures and Tables

**Fig. A:**
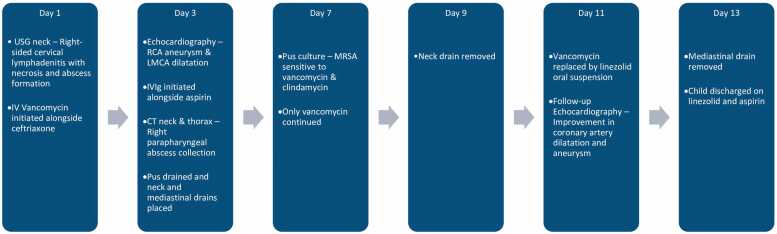
Timeline summarizing the patient’s clinical course, and key interventions during hospitalization

**Fig. B:**
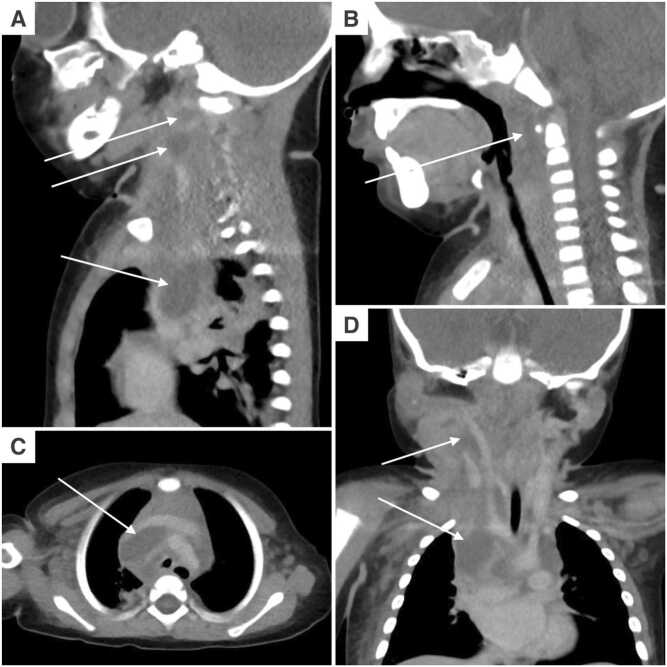
Contrast enhanced neck and thorax CT (A) in sagittal plane showing right parapharyngeal abscess collection with extension into the right carotid space, superior and middle mediastinum; (B) in sagittal plane showing a retropharyngeal abscess (C) in axial plane showing a mediastinal abscess at T4 level (D) in coronal plane showing a right parapharyngeal abscess extending until the mediastinum

**Table 1:** Results of hematological and biochemical investigations in this case

Investigations	Day 1	Day 5	Day 10	Day 12
CRP (mg/L)^*a*^	242	20		9
ESR (mm/h)^*b*^	76	49		
Hb (g/L)^*c*^	75	102	109	101
MCV (fL)^*d*^	58.3	64.3	67.8	66.8
MCHC (g/L)^*e*^	306	309	302	310
WBC (x10^9^/L)^*f*^	35.5	27.3	21.1	17.2
Platelets (x10^9^/L)	292	674	984	1147
Urinary leukocytes/ pus cells (cells/HPF)	1			
Serum albumin (g/L)	28			
ALT (U/L)^*g*^	14			
AST (U/L)^*h*^	17			
Troponin-T (µg/L)	0.00931			
Serum total bilirubin (µmol/L)	3.42			

^*a*^ C-reactive protein; ^*b*^ Erythrocyte sedimentation rate; ^*c*^ Hemoglobin (whole blood); ^*d*^ Mean corpuscular volume; ^*e*^ Mean corpuscular hemoglobin concentration; ^*f*^ White blood cell count; ^*g*^ Alanine aminotransferase; ^*h*^ Aspartate aminotransferase.

## Data Availability

The data that support the findings of this case report are not publicly available due to privacy and ethical restrictions. Relevant clinical information is included within the article. Further details are available from the corresponding author upon reasonable request and with appropriate ethical approvals.
